# ‘We are the change’ - An innovative community-based response to address self-stigma: A pilot study focusing on people living with HIV in Zimbabwe

**DOI:** 10.1371/journal.pone.0210152

**Published:** 2019-02-13

**Authors:** Nadine Ferris France, Stephen H.-F. Macdonald, Ronan R. Conroy, Patrick Chiroro, Deirdre Ni Cheallaigh, Masimba Nyamucheta, Bekezela Mapanda, Godsway Shumba, Dennis Mudede, Elaine Byrne

**Affiliations:** 1 The Work for Change / Irish Forum for Global Health, c/o Department of Epidemiology and Public Health Medicine, Royal College of Surgeons in Ireland, Dublin, Ireland; 2 Department of Epidemiology and Public Health Medicine, Royal College of Surgeons in Ireland, Dublin, Ireland; 3 Impact Research International, Pretoria, South Africa; 4 Trócaire, Maynooth, Co. Kildare, Ireland; 5 Zimbabwe National Network of People Living with HIV (ZNNP+), Harare, Zimbabwe; 6 Independent Consultant to Trócaire, Maynooth, Co. Kildare, Ireland; 7 Connect Zimbabwe Institute of Systemic Therapy (ZIST), Harare, Zimbabwe; 8 RCSI Institute of Leadership, Royal College of Surgeons in Ireland, Dublin, Ireland; Brown University, UNITED STATES

## Abstract

**Introduction:**

Self-stigma–negative self-judgements resulting in shame, worthlessness and self-blame–may play a crucial role in emotional reactions and cause emotional distress among many people living with HIV and other chronic illnesses. Furthermore, self-stigma negatively impacts on self-agency, quality of life, adherence to treatment, and access to services. High levels of self-stigma have been reported across many countries, however few programmes or interventions exist to specifically tackle this phenomenon. This paper reports the findings of a pilot study carried out in Zimbabwe using a programme incorporating “Inquiry-Based Stress Reduction (IBSR): The Work of Byron Katie”–a guided form of self-inquiry which helps users to overcome negative thoughts and beliefs.

**Objectives:**

The primary objective of this uncontrolled pilot study was to examine the potential role of the IBSR intervention in helping people living with HIV to overcome self-stigma and associated states.

**Methods:**

23 people living with HIV (17 Female, 6 male, average age 41 years) were recruited from a local HIV support network, via open call for volunteers. All participants received the intervention, consisting of a 12-week facilitated programme using techniques derived from IBSR: The Work of Byron Katie. Qualitative and quantitative data were collected and analysed pre- and post-programme.

**Results:**

After taking part in the intervention, participants reported significant improvements in factors including self-stigma (1-month follow-up vs baseline *Z* = 2.1, *p* = 0.039; 3-month follow-up vs baseline *Z* = 3.0, *p* = 0.003, n = 23, Wilcoxon Matched Pairs Signed Rank Test) and depression (1mo vs baseline *Z* = 3.7, *p* = <0.001; 3mo vs baseline *Z* = 3.3, *p* = 0.001). Qualitatively, participants reported improvements including lessened fears around disclosure of their HIV status, reduced feelings of life limitations due to HIV, and greater positive mentality. Improvements persisted at three-month follow-up.

**Conclusion:**

With further development and larger comparative studies to confirm effects, the IBSR programme could become a novel tool to enable people living with HIV to support themselves in overcoming self-stigma.

## Introduction

Much has been published about stigma in relation to HIV and AIDS, mental illness, physical deformities, disability and illness [1–7]. However, much less is understood about *self-stigma*, or internalised stigma–influenced by “the fundamental, inflexible, absolute and generalised beliefs that people hold about themselves, others, the world and/ or the future” [8]–that is the stigma imposed upon the self by the self [9–11]. Self-stigma entails negative self-judgements, which at times may result in feelings of shame, worthlessness and self-blame [10,12,13]. The three major mechanisms of stigma at the individual level–enacted stigma, anticipated stigma, and internalised stigma–are highly complex, interrelated phenomena [14–16], the context of which is that of marginalisation where stigma operates as a multidimensional process, with that marginalisation as both a contributor and outcome [17].

It should not in any way be inferred that self-stigma is somehow the “fault” of the individual who experiences it. Stigma does not arise within an individual in isolation. Rather, stigma becomes internalised through exposure to the social, structural, and cultural contexts that feature enacted, anticipated, and perceived stigma [12,17]. Individuals exposed to these contexts naturally absorb the harmful social and community attitudes towards a stigmatised condition [17] and may experience self-stigma without necessarily having been on the receiving end of enacted stigma [6]. It should also not be inferred that tackling self-stigma alone is enough to address the challenge of HIV stigma as a whole. Rather, as a multidimensional phenomenon, HIV stigma must necessarily be addressed via approaches that are targeted to its different levels and manifestations [18,19], with self-stigma being one component of the wider picture [20].

It is clear, however, that detrimental effects can arise as a result of the internalisation of stigmatising beliefs. For example, self-stigma can lead to a sense of isolation and may play a crucial role in the emotional reactions and cause emotional distress to many people living with HIV (PLHIV) [21,22] and other chronic illnesses [23]. Furthermore, self-stigma may exert wide impacts on social interaction, health services utilisation, treatment compliance, and mental health status [21,24–26]. It also has the potential for adverse behavioural effects including not seeking treatment and care services [27]. A study by Earnshaw [28] also reported that people with chronic illness who had self-stigma accessed care less and had a lower quality of life. Furthermore, a study by Scambler and Hopkins on people living with epilepsy [6] also highlighted the fact that nine out of every ten people interviewed admitted to suffering from self-stigma, although only one third could recall having experienced external stigma.

The damaging effects of stigma may also be compounded by restrictive legal and/ or socioeconomic environments and practices that target those living with, or at increased risk of, HIV [29,30]. Such contributing factors are also reported to lead to low self-esteem and threaten self-efficacy of PLHIV [9]. Internalised stigma has also been associated with the development of depressive symptoms [31], and it is likely that self-defacing internal representations are important in predicting long-term coping and positive living [10]. This is especially relevant among PLHIV, where stigma in its many forms acts as a barrier to prevention strategies, access to care, and wellbeing across many dimensions [14,24,27], as well as amplifying inequalities of gender, race and sexuality [17].

There is increasing evidence to show that PLHIV may experience self-stigma more frequently than they receive enacted stigma from other people [32]. For example, in a survey of 2306 adults in the general public in Cape Town, 38% of PLHIV interviewed reported feeling ashamed of their condition as compared to 16% of HIV-negative people who believed that PLHIV should be ashamed of their condition [21]. Additionally, 13% of those interviewed believed that PLHIV did something to deserve it compared to 41% of PLHIV who felt guilty about having been infected with HIV. Importantly, although in this article we focus on self-stigma, the globally high rates of HIV stigma in its various forms should not be ignored. The 2014 Analytical Report of the Ukraine People Living With HIV Stigma Index showed that 40% of respondents had experienced external stigma, whereas 82% had experienced self-stigma [33]. In a study across nine countries of the Asia Pacific region internalised stigma in the form of shame, guilt and self-loathing occurred at high levels, ranging from 54% in Sri Lanka reporting feeling ashamed of their HIV status to 75% in Pakistan [32]. Additionally, the 2014 HIV Stigma Index for South Africa found that among 10,473 respondents, 43% reported experiencing internalised stigma, with 36% experiencing external stigma [34].

Poor health outcomes for people living with chronic illnesses, such as HIV, diabetes, cancer, may be linked with lack of psychosocial supports, and low quality of life [35–38], and conversely the presence of adequate support is associated with improved health care access and coping with multidimensional aspects of living with HIV and related stigma [39–42]. Furthermore, depression and social support are also among the most frequently and consistently reported factors influencing quality of life among PLHIV [43], and Bhatia *et al*. have found that newly-diagnosed PLHIV are at high risk for depression and poor linkage to care [44]. Approaches that target self-stigma are therefore likely to benefit psychological function and wellbeing among PLHIV [45]. However, despite the range of interventions that exist targeting external stigma [18,46], there is a paucity of measures that target and provide individuals with tools to manage and overcome *self-*stigma [10]. Furthermore, even in studies where high levels of self-stigma have been reported, as with the Asia-Pacific Stigma Index nine-country study, no specific recommendations for interventions on self-stigma are made [32]. External and internal stigma equally have the opportunity and potential to create harm, and both are components of the wider socially- and structurally-created contexts that lead to marginalisation of PLHIV [17]. Multidimensional approaches are therefore likely to yield the best outcomes, and the application of tailored interventions addressing both enacted stigma and internalised stigma, with the aim of improving treatment uptake and adherence, is supported by a growing body of work [19,20]. There is also a broader need to increase provision of frameworks that enable practitioners to assess the social outcomes of interventions, given the breadth of influence that HIV exerts across all aspects of the lives of PLHIV [47].

Brown and Vanable [48], reviewing the stress-management intervention literature for PLHIV, concluded that there is evidence demonstrating that stress-management interventions reduce psychological distress and improve psychosocial functioning. These interventions include cognitive behavioural approaches, as well as meditation, mindfulness, and relaxation-based stress management approaches. Kalichman and Simbayi [49] acknowledge that approaches are needed that go beyond mass education and that work within more ingrained belief systems. In their 2011 systematic review of HIV stigma reduction interventions, Sengupta *et al*. also highlighted the need to incorporate research that builds understanding of stigma, and its drivers, to help create targeted interventions [18].

In this paper, we focus specifically on an intervention to address self-stigma among PLHIV in Zimbabwe, but similar interventions could likely be designed, and similar outcomes achieved, in relation to other chronic illness and diseases. The HIV prevalence in Zimbabwe for 2016 (most recent data at time of writing) is estimated to be 13.5%, with around 1.4 Million PLHIV, approximately 720,000 of whom are women aged 15 years and up [50]. HIV Stigma continues to affect the lives of Zimbabwean PLHIV, with 65.5% of the 1,905 respondents to the 2014 Zimbabwe HIV Stigma Index reporting that they had experienced HIV stigma in one form or another. Among these, social exclusion and physical harassment or threats were the most common (21% and 19% of respondents, respectively). Within this context, internalised stigma also occurred, with 18.9% experiencing guilt or low self-esteem due to their HIV status, 17.9% experiencing self-blame, and 16.7% feeling ashamed. Fear of received stigma was also prevalent, with 37.2% afraid of being gossiped about, 17% afraid of sexual rejection, and 15.4% afraid of being verbally insulted [51].

In light of the persistence of self-stigma, and the lack of proven interventions to address it, it is necessary to seek measures that support individuals to navigate it, particularly at a local level, adapted to the communities in which those most affected by self-stigma live. Participation of communities and local stakeholders has been identified as both a need and an enabler of comprehensive HIV prevention, treatment, and support in several settings, including Zimbabwe [42,52,53].

The multidimensional nature of stigma necessitates development of approaches that operate at different levels. As an addition to that toolkit of interventions, we sought to examine the potential role of a stress reduction intervention, “Inquiry-Based Stress Reduction (IBSR): The Work of Byron Katie”, to help PLHIV to navigate self-stigma utilising a protocol derived from methods used in “The Work of Byron Katie” [54,55], and adapted for use in the Zimbabwean context. IBSR: The Work of Byron Katie is a facilitated meditative technique of self-inquiry used to manage stress and other negative thoughts, and this method has been previously demonstrated to positively affect psychological scales and mental health [56], as well as reducing psychopathologic symptoms such as depression, anxiety, and obsessive-compulsiveness [57] in non-clinical groups. Elsewhere, the stress reduction approach using other interventions such as mindfulness has demonstrated positive effects in improving psychological status and reducing impact of HIV treatment side effects [58,59]. The first IBSR: The Work of Byron Katie pilot clinical trial has been completed in breast cancer survivors in Tel Aviv, reporting significantly lower levels of fatigue and improved sleep quality, as well as physical, social, familial, emotional and functional wellbeing [60]. Furthermore, IBSR: The Work of Byron Katie has also recently been shown to be effective in improving psychological symptoms and quality of life in a pilot clinical study by Smernoff *et al*. in a non-clinical setting [61], and it has also been used in a randomised controlled trial to examine the effect on psychological wellbeing among oncologically-healthy carriers of the BRCA1/2 mutation [62]. Similar interventions can potentially be a cost-effective means to control stress and improve quality of life among the general population [63] (also reviewed in [64]), and have also been shown to create reductions in psychological distress, sleep disturbance and fatigue, as well as increases in positive psychological outcomes, in cancer patients–reviewed in Rouleau *et al*. [65].

IBSR: The Work of Byron Katie has not previously been used to address HIV self-stigma, and has not been used in Zimbabwe. However, it was chosen opportunistically in this case since NFF and DNC are certified facilitators of the Work for Change, and both have past exposure and familiarity with IBSR as well as working in the area of stigma and PLHIV. Additionally, there was an opportunity afforded by the existing working relationship between Trócaire in Zimbabwe with the CONNECT Zimbabwe Institute of Systemic Therapy (ZIST) and the Zimbabwean National Network of PLHIV (ZNNP+). For these reasons, we decided to explore the potential of IBSR as a means of overcoming self-stigma among people living with HIV in the Zimbabwean context, taking into account its potential as a psychosocial intervention as demonstrated by others, as well as the thematic alignment of its techniques with the professional counseling expertise of staff at CONNECT ZIST.

This pilot study was designed to test the possible influences and acceptability of IBSR: The Work of Byron Katie in the management of HIV self-stigma, and the following research questions were formulated:

1. Does the intervention using IBSR: The Work of Byron Katie have the potential to influence self-stigma?
2. Does the intervention have potential to influence stigma-associated states, such as shame, fear around disclosure, and depression?
3. Does the intervention have potential to influence other areas of participants’ lives beyond HIV self-stigma?

Prior to commencing this pilot study, no local facilitators existed, therefore we conducted the intervention using internationally-certified facilitators sourced from outside Zimbabwe. This helped ensure that the intervention could be delivered with sufficient consistency and quality. We intended that this pilot study would help inform further studies that would a) explore any positive effects that might arise following to the intervention, and b) further refine the intervention itself. It would also lay the groundwork to create a programme to train local facilitators sourced from Zimbabwe and trained in the same manner, and to the same standard, as the internationally-certified facilitators, moving towards an entirely Zimbabwean-led programme of support for PLHIV to deal with self-stigma.

### The intervention: Inquiry-Based Stress Reduction: The Work of Byron Katie

**Fig 1:**
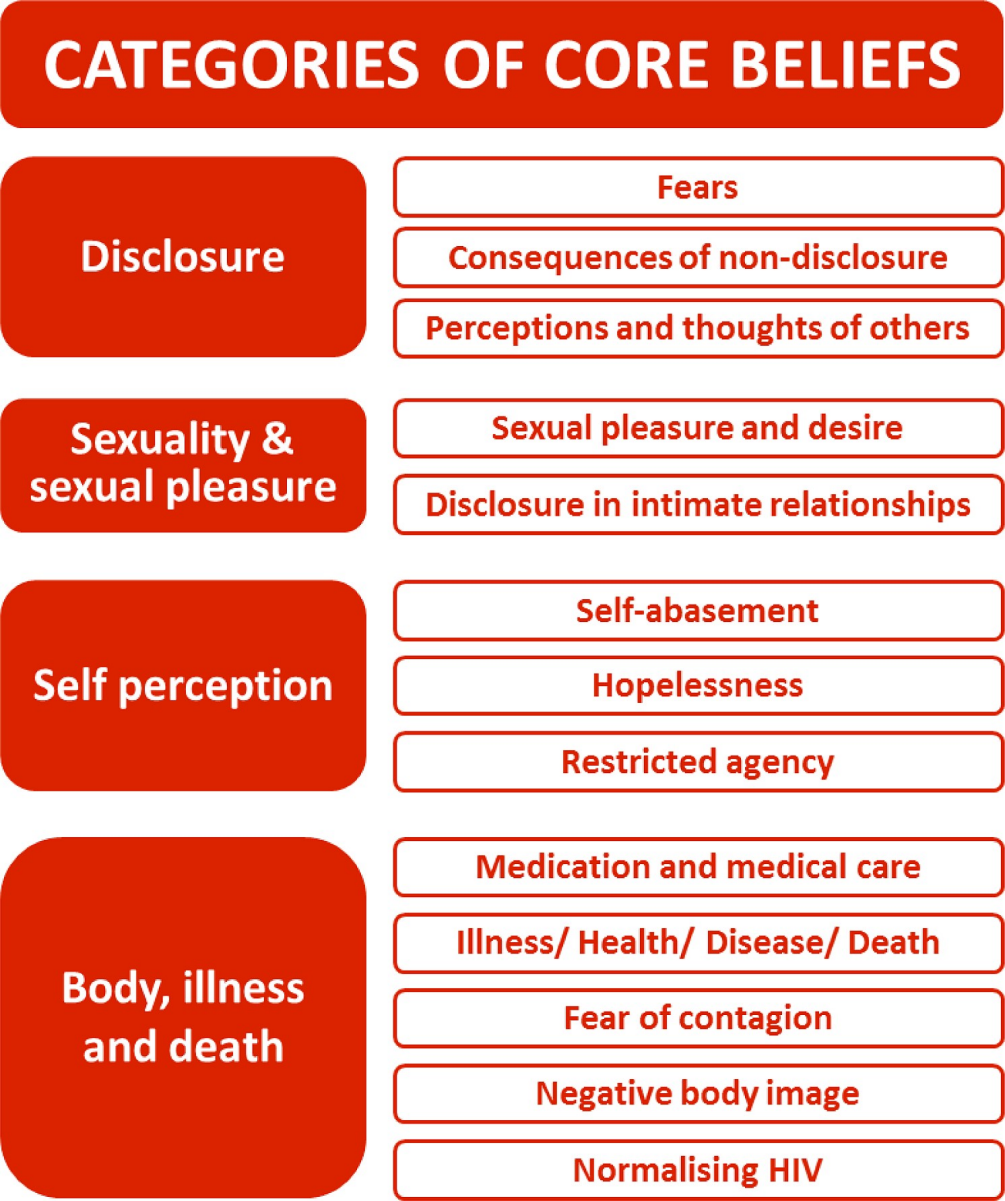
Core beliefs which contribute to HIV self-stigma. Showing core beliefs contributing to HIV self-stigma among persons living with HIV (unshaded boxes), which fall broadly across four categories (shaded boxes): Disclosure; Sexuality & Sexual Pleasure; Self-perception; and Body, illness and death. (Reproduced from Ferris France *et al*., 2015 [12] (Swiss Med Wkly. 2015;145:w14113) under a CC-BY License with permission from EMH Swiss Medical Publishers Ltd., original copyright 2015).

Our findings and recommendations from a formative qualitative study identifying core beliefs underlying self-stigma among PLHIV in Ireland [12] informed the design of an operations research intervention pilot study that used a 12-week programme using techniques from IBSR: The Work of Byron Katie. Based on the research findings, a curriculum was designed to support participants to question and work through self-stigmatising beliefs (see Fig 1, reproduced from [12]). Technical support and curriculum development was carried out by International Certified Facilitators of the Work of Byron Katie, coordinated by The Work for Change organisation (http://www.theworkforchange.org). International Certified Facilitators have previously completed certification with The Worldwide Institute for the Work (http://www.instituteforthework.com) conducted over 2–5 years, consisting of 620 hours of study incorporating face-to-face workshops, facilitated remote classes, client contact hours, self-inquiry, and peer and mentor methods.

The intervention curriculum was designed to start with a number of universal stressful thoughts about being HIV positive and from there to work systematically through more acute self-stigmatising core beliefs at a deeper level each week. During the process, participants were encouraged to identify, and inquire about, their stressful thoughts regarding shame, HIV status disclosure and other HIV-related issues, sexuality, sexual pleasure and intimate relationships, self-judgements and body, illness and death. The intervention aimed to improve management of specific emotional and psychological symptoms (relating to self-worth, stress, anxiety, depression, fears), and physical symptoms such as fatigue, and managing other medication-related side effects.

Through the use of self-inquiry, subjects were taught to increase awareness of their thoughts and feelings, and to observe their emotional and physical responses during situations perceived by them as stressful. The participant appraises their thoughts using the following questions: (1) *Is it true*?; (2) *Can I absolutely know that it is true*?; (3) *How do I react when I believe that thought*?; and (4) *Who would I be without the thought*? This process is meditative and reflexive, and the participant is guided to search for the true and genuine answers to the four questions with no certain agenda or censoring of thoughts. In this way, the participants become more aware of their self-stigmatising thoughts as they occur. The final stage involves ‘turnarounds’, where by turning the original thought or belief around in different ways–to the self, to the other and to the opposite, the participant experiences different interpretations of their own reality. By finding three genuine examples where each ‘turnaround’ is as true as the original thought, participants gain understanding of the power of stressful thoughts, and the ability to question these thoughts. This can help in managing their own thoughts to better cope in what had previously been highly stressful situations [54,66].

In doing so, subjects took an active role in investigating their stressful thoughts, regulating their stress and managing symptoms and emotions, thus enabling them to cope better with the distress related to self-stigma as a result of HIV. Two local facilitators worked with international certified facilitators to deliver and adapt the programme with two groups of participants (23 in total) drawn from support groups within ZNNP+. The participants took part in 12 four-hour group sessions run over 12 weeks at CONNECT ZIST, a weekly one-hour individual session with a facilitator also at CONNECT ZIST, as well as homework. Tools from IBSR: The Work of Byron Katie were used throughout the course to support participants to identify and list their beliefs and thoughts. A participant was considered active if they were present in at least 90% of the group and individual sessions. Participation was not incentivised.

## Materials and methods

### Ethical approval

Participants took part in this study freely and their involvement was not incentivised in any way. Prior to commencing the study, participants were asked to give written informed consent, and they were free to withdraw from the study at any time. Ethical approval for this study was granted by the Medical Research Council (MRC) of Zimbabwe (approval number MRCZ/A/1782). The Royal College of Surgeons in Ireland (RCSI), Trócaire, and CONNECT ZIST, did not themselves require separate ethics submissions, and agreed to collaborate subject to the granting of the ethical approval by the MRC of Zimbabwe.

### Participant recruitment

Participant recruitment commenced on 28 October 2013 and ended on 21 February 2014. Follow-up ended on 30 August 2014.

### Clinical trial registration

This study was retrospectively registered as a clinical trial in the ISRCTN Registry (https://www.isrctn.com/), registration number ISRCTN17045085 (https://doi.org/10.1186/ISRCTN17045085) We had not registered this study as a clinical trial before commencement due to unfamiliarity with guidance around classification of trials. Nonetheless, ethical requirements by the Medical Research Council of Zimbabwe, our funders Trócaire, and partner organisations, were fulfilled throughout the study, with full protection (confidentiality, anonymity) and support given to the study participants, and all data has been reported faithfully without omission or bias towards positive or negative outcomes. There are currently no ongoing trials related to this study.

### Adaptation of the intervention for the Zimbabwean context

This was the first trial of the IBSR: The Work of Byron Katie methodology to be conducted in Zimbabwe, as well as in the context of HIV self-stigma. The adaptation of the intervention and associated materials were greatly facilitated by involvement of the CONNECT Zimbabwe Institute of Systemic Therapy (http://www.connect.co.zw/). CONNECT is a welfare organisation established in 1983, registered with the Zimbabwean Department of Social Welfare in 1985, which conducts community outreach, counselling, training, and research incorporating psychosocial interventions around mental health care for people living with HIV and other conditions. These links were critical in facilitating participant recruitment, as well as logistical aspects of the project. The intervention was adapted for use in Zimbabwe through collaboration between CONNECT, Trócaire, The Work for Change, and the RCSI research team. To ensure truly local adaptation, two Zimbabwean PLHIV were supported to attend a nine-day IBSR: The Work of Byron Katie training workshop in Germany, who then worked together with International Certified Facilitators in designing the curriculum. Systematic debriefings following delivery of each session with local and international facilitators ensured adaptation of the sessions to the local context where some modules were created to respond to cultural beliefs that emerged during discussions. Local Zimbabwean staff also role-played using the intervention tools to ensure suitability, and were present throughout the intervention workshops to assist with translation of uncommon phrases, and to help with clarification of materials by adapting them into the local idiom.

### Study design

**Fig 2:**
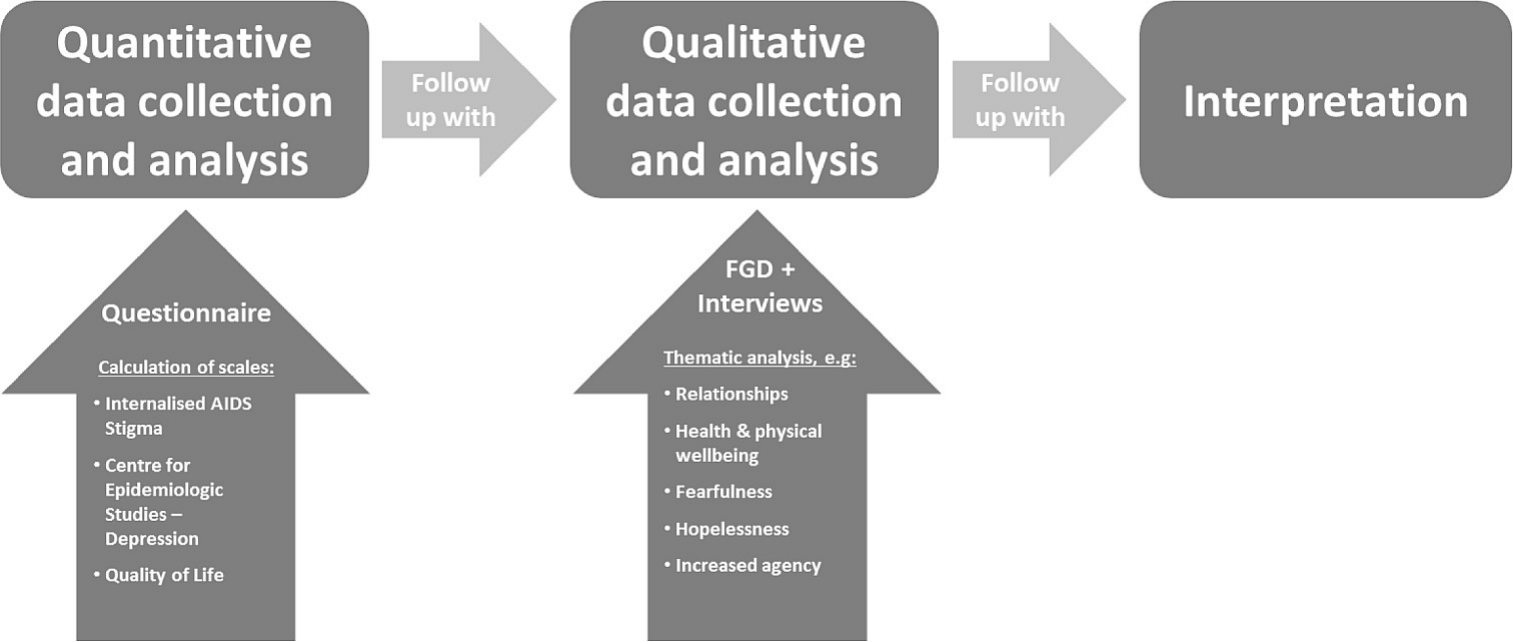
Sequential mixed methods study design. Initial data collection and analysis of quantitative scales was carried out using a questionnaire delivered to participants, incorporating items from the Internalised AIDS Stigma scale; Centre for Epidemiologic Studies–Depression scale; and HIV/AIDS Targeted Quality of Life Scale. This was followed by qualitative data collection and thematic analysis from Focus Group Discussions and one-on-one Interviews.

Since this was the first time the intervention had been used against HIV self-stigma in Zimbabwe, we adopted a sequential explanatory mixed methods design for this study [67] (Fig 2), in order to gain a deeper understanding of any possible influences than could be gained by quantitative measurement alone. Our dual aims were a) to measure the *quantity and direction* of changes in self-stigma and associated states as a result of the intervention, and b) to investigate possible reasons *why* these changes occurred, and what other areas of participants’ lives might be affected. Addressing the first aim, we used quantitative questionnaires that incorporated previously-validated and well-established scales to measure HIV self-stigma, depression, and HIV-associated quality of life. In parallel to this, to achieve the second aim we employed semi-structured qualitative interviews and focus group discussions to gain understanding of the drivers of self-stigma among the participants and the ways in which they might have been influenced by the intervention, and uncover any unexpected areas which could be targeted in future interventions against HIV self-stigma.

### Data collection

Questionnaires, FGDs and interviews were administered by members of the research team (NFF, GS) in private locations at CONNECT ZIST (the same facility where the intervention took place). Intervention facilitators were an equal balance of female and male, and all had previously worked in qualitative and/ or quantitative research with PLHIV, in academic and NGO contexts. Researchers administered a structured questionnaire to all participants on three occasions: a day before the course, 1 month after the course and at 3-month follow-up. (For complete questionnaire see Supporting Material S1 File) Two separate focus group discussions (FGDs) (average duration 1h 32min.) were held on the day before, and a month after the intervention. Each FGD aimed to access an equal number of female and male participants. Six key informant interviews (average duration 26 min.) were also conducted with participants post-course. The FGDs and interviews were conducted in a semi-structured manner, guided by a set of open-ended questions such as “If you had to describe what you got out of this course, what would you say?”, with minimal interviewer interjections except to clarify or probe for greater detail. (The semi-structured FGD and interview guides are reproduced in full in Supporting Material S2 File) The interview / FGD members were drawn from the overall pool of 23 participants, and participants volunteered themselves for individual interviews following a general call, with no incentivization or pressure to take part. The interview participants all also took part in the FGDs. None of the interviewees and focus group members requested transcripts after participating. All interviews and FGDs were conducted in English. Data were anonymised after collection, with individuals only known by their participant number. Team members performing analysis were blind to the identity of the participants.

### Quantitative analysis

The structured questionnaire included scales and components of the Internalised AIDS Stigma Scale (IASS) [10] (12 items), the Center for Epidemiologic Studies Depression Scale (CES-D) [68] (20 items), and the HIV/AIDS Targeted Quality of Life Scale [69] (28 items), to measure mood, perceived stress, and quality of life. Participants gave responses to questions on Likert-type scales, for example ranging from “strongly disagree” to “strongly agree”. Responses were then coded numerically, and scale scores were calculated as the average of the items in the scale. The responses were matched by individual and analysed longitudinally. No scores were missing at any time point. Results of participants’ responses (raw data can be found in Supporting Material S3 File, and calculations in the Supporting Material S4 File) on these scales were calculated using STATA version 15 (Stata Corp, Texas, USA). Changes in score, together with their interquartile range (IQR) are presented. Changes in scores between baseline and 1- and 3-month follow-up were analysed using the Wilcoxon matched-pairs signed rank test. As a measure of effect size, we calculated *r*, based on the *Z* value of the Wilcoxon test, using the transformation *r* = *Z*÷√N.

### Qualitative analysis

The FGDs and interviews explored the concepts of stigma, self-stigma, depression, stress and quality of life, as well as the perceived triggers and consequences thereof on participants’ lived experiences. Discussions and interviews were recorded with prior informed consent, transcribed, then analysed using a semi-inductive iterative coding strategy in QDAMiner Lite version 1.4.1 (Provalis Research, Montreal, Canada), including themes derived from our previous work [12], and new themes as they arose during coding. Three members of the research team (SM, NFF, EB) independently analysed transcripts until saturation was reached, then met to review for consistency and finalise the list of themes. For clarity in analysing the participants’ experiences of external and self-stigma, we chose to arrange categories and themes to distinguish internal challenges due to emotions, feelings, and anticipation of events, as well as tangible external experiences and outcomes. As a result, some categories appeared to overlap thematically but were kept distinct for the purposes of our analysis. For example, we coded *Negative Perceptions of Others* (Category) > *Perceptions of PLHIV* (Theme), where participants reported experiencing stigma or witnessing other people relating negative preconceptions about PLHIV. This was in contrast to *Fearfulness* (Category) > *Disclosure* (Theme) > *Afraid of what others will think* (Sub-theme), in which participants reported that they themselves had felt afraid of the consequences if their HIV status were to become known. (For full list of themes, see Supporting Material S5 File)

### Participants

23 individuals volunteered for this study following an open call for participants between October 2013 and February 2014. All were members of Harare-based support groups which were part of the ZNNP+ network. At least 24h prior to taking part, participants were given an information leaflet outlining the purpose of the research, assurance of anonymity, confidentiality, and the right to withdraw at any time. 17 participants were female; 6 were male, and average age was 41 years, ranging from 25–54. Due to the stigmatised nature of minority sexual orientations in Zimbabwe, we did not ask participants to disclose their sexual orientations during recruitment, nor did we ask if they had acquired HIV through heterosexual or homosexual activity. Further participant details are included in Table 1. All 23 participants received the intervention, and all took part until completion (all participants took part in all required sessions), commencing in late February 2014, with final follow-up in August 2014. None were lost to follow-up. Participants were not previously known to the researchers before taking part in the study. Individuals were approached to take part in FGDs and interviews opportunistically, and none declined to participate. Eligibility criteria: Participants included were: 1) Individuals living with a positive HIV diagnosis for longer than three months; 2) Over 18 years of age; 3) Able to speak English with sufficient fluency to take part in group discussions; 4) Willing to provide informed consent and sign an informed consent form. Individuals excluded were: 1) Individuals with unknown HIV status; 2) Individuals diagnosed with HIV in the last three months; 3) Individuals currently undergoing psychotherapy treatment.

**Table 1:** Characteristics of study participants. Showing participants’ age, sex, partnership, education, and employment status, and whether or not they had children.

***Age (years)***	
Mean	**41**
Range	**25–54**
***Sex***	
Female	**17**
Male	**6**
***Antiretroviral (ARV) use (years)***	
Average no. of years on ARVs	**6.8**
Range	**3–13**
***Living with stable partner***	
Yes	**15**
No	**8**
***Currently employed***	
Yes	**7**
No	**16**
***Completed form 4 education***	
Yes	**15**
No	**8**
***Has children***	
Yes	**23**
No	**0**

## Results

### Participant-reported improvement of self-stigma and associated measures post-intervention

Significant changes across a number of measures relating to self-stigma were reported by participants at follow-up, after taking part in the intervention. Follow-up scores for self-stigma were significantly improved (i.e. self-stigma was reduced) at both 1-month (*Z* = 2.1, *p* = 0.039) and 3-month (*Z* = 3.0, *p* = 0.003) timepoints compared to baseline. A similar trend was also recorded for depression at 1-month (*Z* = 3.7, *p* = <0.001) and 3-month (*Z* = 3.3, *p* = 0.001) timepoints. Daily activities were also improved at 1-month (*Z* = 2.8, *p* = 0.005) and 3-month (*Z* = 2.8, *p* = 0.004) timepoints. View of the future was not significantly different at the 1-month timepoint (*Z* = 1.3, *p* = 0.198), but was significantly improved at 3-months (*Z* = 2.1, *p* = 0.033). Feelings about being HIV-positive also were not significantly improved at the 1-month timepoint (*Z* = 1.0, *p* = 0.323), but were significantly improved at 3-months (*Z* = 2.1, *p* = 0.038). Fears around disclosure of HIV status were significantly reduced at both 1-month (*Z* = 2.0, *p* = 0.046) and 3-month (*Z* = 2.0, *p* = 0.047). Life satisfaction was significantly improved at the 1-month timepoint (*Z* = 2.4, *p* = 0.017), but not at 3-months (*Z* = 1.7, *p* = 0.098). No significant differences compared to baseline were reported for social support (total) (1-month *Z* = 0.5 *p* = 0.615; 3-month *Z* = 1.5, *p* = 0.132), social support (intimacy) (1-month *Z* = 0.61, *p* = 0.543; 3-month *Z* = 1.2, *p* = 0.235), or social support (help from others) (1-month *Z* = 0.8, *p* = 0.402; 3-month *Z* = 1.5, *p* = 0.144) (n = 23; results summarised alongside median difference, IQR, and effect size, in Table 2).

**Table 2:** Quantitative results from self-stigma and associated measures questionnaire post-intervention. Showing baseline median, median difference and interquartile range, Z-score, effect size, and significance, between baseline and 1- or 3-month follow-up timepoints, for participants’ self-reported scores in scales for Self-stigma, Depression, Daily activities, View of the future, Feelings around being HIV+, Fears around disclosure, Life satisfaction, Social support (total), Social support (intimacy), and Social support (help from others) (n = 23).

		Baseline– 1 month				Baseline– 3 month			
Outcome measure	Baseline median	Median difference (IQR)	*Z*	Effect size (r)	sig	Median difference (IQR)	*Z*	Effect size (r)	sig
Self-stigma	0.83	0.17 (0–0.8)	2.1	0.43	0.039	0.5 (0–0.8)	3.0	0.62	0.003
Depression	1.25	0.3 (0.1–0.7)	3.7	0.77	<0.001	0.3 (0.1–0.7)	3.3	0.67	0.001
Daily activities	1.4	0.4 (0–1)	2.8	0.59	0.005	0.4 (0–1.2)	2.8	0.60	0.004
View of the future	1.67	0.3 (-0.3–0.7)	1.3	0.27	0.198	0.3 (-0.3–1.3)	2.1	0.44	0.033
Feelings around being HIV+	0	0 (0–1)	1.0	0.21	0.323	0 (0–1)	2.1	0.43	0.038
Fears around disclosure	0.4	0 (0–0.4)	2.0	0.42	0.046	0.2 (0–0.6)	2.0	0.41	0.047
Life satisfaction	2.75	0.75 (-0.1–1.3)	2.4	0.50	0.017	0.5 (-0.2–1.3)	1.7	0.35	0.098
Social support (total)	2.25	0.1 (-0.17–0.33)	0.5	0.11	0.615	0 (-0.17–0.6)	1.5	0.31	0.132
Social support (intimacy)	2.4	0 (-0.2–0.4)	0.61	0.13	0.543	0 (-0.2–0.6)	1.2	0.25	0.235
Social support (help from others)	2.14	0.1 (-0.3–0.3)	0.8	0.17	0.402	0.1 (-0.6–0.2)	1.5	0.30	0.144

The quantitative data from the questionnaires showed that after taking part in the IBSR: The Work of Byron Katie programme, participants reported significant and sustained changes across several areas of their lives. However, whilst this clearly indicated the size and direction of the change, the scales used did not capture information regarding the circumstances, as well as the underlying drivers of such change. To address this, and to gain deeper understanding of the participants' experience of living with HIV, the FGDs and one-to-one interviews were analysed to capture a greater understanding of the complexities behind the data. In combination with post-course FGDs and interviews, this analysis allowed for the exploration of the wider context surrounding living with HIV, and the underlying sources of stress, stigma, self-stigma and associated negative outcomes, with which participants had been living, and often struggling to cope with, for many years.

### Self-stigma and negative influences on participants' lives: Fearfulness, shame, guilt, hopelessness, worthlessness, restricted agency, and negative perceptions of others

The narrative recounted by participants regarding HIV before the intervention, the circumstances surrounding their infection, and the possible influence this would have on their lives thereafter, was overwhelmingly negative. Participants’ responses broadly fell into categories including *Fearfulness*, *Shame*, *Guilt*, *Hopelessness*, *Worthlessness*, *Restricted Agency*, *and Negative perceptions of others*.

The first category, *Fearfulness*, was experienced in a number of ways–for example, fear surrounding disclosure of one’s HIV status to others, fear for their dependents in the event of their death, and fear for their personal health:

*“Before I attended the course*, *I thought that I may die soon. I was afraid of the side effects of ARVs. I disliked being positive.” Post-course Interview Participant A10*

A fear of suffering from other diseases which could accompany, or be worsened by, HIV was also reported by the participants. It became clear that they obviously had access to knowledge surrounding the broader health risks presented by living with HIV, but they also lacked mental tools or training to cope with the accompanying psychological burden:

*“When I fall sick*, *I think the worst*. *For example*, *when I have rash on my body*, *I think its Kaposi Sarcoma* … *When you start coughing*, *you think its TB* … *Whenever I fall sick*, *I think the worst*.*”* Pre-course Focus Group A

Some also spoke of experiencing several dimensions of *Shame* and *Guilt*–for example being ashamed of themselves as a person living with HIV, or over becoming HIV positive, as well as blaming those who had infected them, with some adding a negative dimension to ordinarily-positive life events:

*“When I tested positive*, *I was ashamed of myself. I blamed myself for getting married.” Pre-course Focus Group B*

Within the category of *Hopelessness*, some participants reported feeling that their life was effectively over upon receiving their HIV positive diagnosis:

*“After seeing that the results were positive*, *I felt it was the end of my life. I felt being vulnerable and being manipulated … The moment I was alone I became afraid, fearing the unknown … I was staying at home always, not doing anything… I told myself that I will not buy any new clothes. I didn’t want my relatives to take away my possessions upon my death.” Pre-course Focus Group A*

Some participants also reported holding negative beliefs about themselves, which fell into the broader category of *Worthlessness*, for example lack of self-worth, and which contributed to *Restricted agency*:

*“I thought I was useless*. *Why should I go on and be educated when I’m [HIV] positive? I didn’t think I will live longer. It was like a death sentence to me. I just didn’t think there was life after being tested positive.” Pre-course Focus Group A*

Participants also often spoke of their sense of loss after their HIV diagnosis, describing negative situations that being HIV positive had caused. This in turn had perpetuated their sense of self-stigma:

*“I tested positive in 2005*. *I lost everything that I had*. *I lost my beloved wife and son*. *I lost my job*. *Life was not easy*. *My in-laws took my children away from me*. *I just started staying alone*. *I suffered feelings of shame*, *guilt and loneliness*.*”* Pre-course Focus Group B

Participants also spoke of the external stigma that they had received, which broadly fell into the category of *Negative perceptions of others*–this often manifested as negative perceptions of HIV, or those living with HIV. Some participants had believed that a diagnosis of HIV meant that life was effectively over, and this belief was also held by some families, resulting in an overarching attitude of pessimism towards those living with HIV:

*“One of my family members started isolating me from the family discussions*. *She was saying I was HIV positive and not capable of taking part in the discussions*. *It was as if the virus had affected my brain and I could no longer think or contribute anything*.*”* Pre-course Focus Group B

Commonly-held perceptions of the source of an HIV infection also coloured interactions of others with the participants following a diagnosis of HIV:

*“When I tested positive in 2008 and told my husband the results*, *he started throwing things everywhere in the house, calling me a prostitute.” Pre-course Focus Group B*

Participants frequently reported that the impact of their HIV diagnosis extended beyond specific worries about physical health and dying: here, far more complex situations were reported, often exerting a significant detrimental effect on family and social lives. In particular, the reactions of family or social groups to an individual's HIV status were frequently negative and disowning, rather than supportive:

*“My sister’s husband couldn’t understand my situation and refused to keep me with my child*. *He said it was your fault to be HIV positive*. *I went on to stay with someone who was not my relative*.*”* Pre-course Focus Group B

In addition to the seriousness of HIV/AIDS itself, often commonly-held (and frequently inaccurate) perceptions of HIV, and those who carry it, also coloured interactions of others with the participants following a diagnosis of HIV:

*“When I tested positive in 2008 and told my husband the results*, *he started throwing things everywhere in the house*, *calling me a prostitute*.*”* Pre-course Focus Group B

Participants reported that following HIV diagnosis, many aspects of their lives were subsequently impacted, contributing to self-stigma and stress, which had become significant moderators of self-perception, agency, and outlook. Whilst arising from many different sources, the outcome was consistently an adverse mental state which contributed to limitation on that individual's personal agency or ability to live in a manner that sustained wellbeing:

*“I would always occupy myself with stressful thoughts*.*” Post-course Interview Participant A11*

### Possible influences of the intervention on self-stigma and personal wellbeing: Change and coping strategies

When speaking of the possible influences in their lives following the intervention, participant responses largely fell into the theme of *Change*, referring to multiple areas of their lives which had improved, and *Coping strategies*, encompassing the mechanisms and activities which were helpful in overcoming self-stigma, and building self-esteem and wellbeing. Examples of these included building acceptance of one’s HIV positive status, and growing self-support such as taking on a positive mentality and overcoming negative thoughts.

Complementing the quantitative measures which indicated significant and lasting improvements in participants' wellbeing, a common qualitatively-reported outcome was that taking part in the intervention had fostered positive change throughout participants’ lives as a whole:

*“My life or the way I conduct myself should tell that I have been through this programme*. *People who live with me should see that my life has changed*. *They should see the change in me*.*”* Post-course Interview Participant A11

The post-intervention interviews and discussions yielded further detail regarding the circumstances surrounding these changes. Improvements had occurred across many areas, ranging from self-perception, personal appearance and self-care, through to interactions with family, colleagues and friends, as well as changes in outlook surrounding living with HIV. In particular, participants reported the realisation that overcoming self-stigma was an important step towards being able to mitigate stigma received from others:

*“I learnt that the moment I worry about what people think about me*, *I am stigmatising myself*. *By stigmatising myself*, *I withdraw*. *When I withdraw*, *I am now allowing people to stigmatise me because I will be watching every movement that they will be doing*. *Yet it’s not them doing it to me but it would be me doing it to myself*. *Through doing ‘The work’*, *I feel that I am able to mix and mingle with anyone*. *I am no longer worried about what people think or say about me because the moment I do that I will be stigmatising myself*. *I now have a very free mind and I am now an outgoing person*.*”* Post-course Interview B6

Prior to taking part in the intervention, many perceived themselves to be 'different' or separate from others due to their HIV status, and as a result felt unable to view themselves, or be viewed, as 'normal'. Following the intervention however, a theme of acceptance was reported, which helped in coping with difficult internal thoughts:

*“I have learned to accept my thoughts with understanding*. *The stressful thoughts that I have are not different from what other people are experiencing*.*”* Post-course Focus Group A

When speaking about difficulties encountered during interactions with others resulting from living with HIV, it was evident that many of the participants experienced shame, or worries about what others thought of them as PLHIV, however the intervention had enabled them to regain their sense of self-confidence:

*“I used to be ashamed of myself*. *I didn’t have confidence of what I was doing*. *Because of this training I am no longer ashamed of myself and the fact that I am [HIV] positive*.*”* Post-course Interview B4

Diminished feelings of shame and self-limitation were also accompanied by an enhanced feeling of self-worth as well as renewed self-agency, where participants reported that they now felt they could live in the same manner as those who are HIV negative:

*“I have realised that with or without HIV*, *I am able to do what other people who are not HIV positive can do*. *I have discovered that I have got no limitations*.*”* Post-course Focus Group B

Several of the participants had described situations prior to the intervention where they felt uncomfortable disclosing their HIV status to others, however having taken part in the course, they had become significantly empowered to discuss such issues and were more comfortable in disclosure–in some cases experiencing unexpected positive outcomes:

*“I used to find it difficult to disclose my status to some of my relatives*. *Due to the course*, *I have disclosed my status to those relatives*. *To my surprise*, *what I thought would be their reaction was totally different from what they did*. *I thought that some of them would be shocked and they would isolate me but nothing changed and their love for me has increased*. *I no longer have a problem of disclosing my status even during social forums that I normally attend*.*”* Post-course interview Participant B9

When speaking of education and knowledge following the intervention, participants' responses were not limited to the benefit gained in dealing with internal thoughts: techniques and knowledge from the programme had been brought into a wide range of areas throughout their lives, for example improved communications with family members, and reduced social isolation. Furthermore, participants reported the intention to use the new knowledge gained to support other PLHIV. It was apparent that the intervention had presented them with a valued tool that potentially enabled them to manage the effects of their self-stigma, and in doing so created a sense of empowerment:

*“I can deliver this programme in support groups for people living with HIV using the vernacular language*. *The course can also be delivered in schools using the English language* … *I can deliver the programme well to the women's group at my church*. *I conduct sessions on positive living at my workplace and I can use that as an opportunity to share what I have learned in the IBSR course*.*”* Post-course Interview Participant A4

A further common theme among participants' responses was a shift towards a more positive mentality. Whereas prior to the intervention, many reported pessimism and a lack of hope for the future, the post-course interviews and focus groups presented a more positively-framed outlook:

*“I got infected in 1993 but I am in 2013*, *which is exactly 20 years living with the virus*. *Now*, *I think I will even live longer because I have learned to appreciate myself* … *I now look at myself better than what I used to do before*. *I look at myself as just a person with any other disease such as diabetes or cancer*. *Right now*, *I am very healthy and I don’t have any complaints*.*”* Post-course Interview Participant A11

All of the situations described by the participants were impacted upon by HIV, yet there was a paucity of reports of specific difficulties due to medication, actual HIV-related illness, or access to medical services. Instead, participants reported limitations caused by self- and perceived stigma, and the far-reaching consequences thereof, which were somewhat mitigated following the intervention:

*“I now feel very comfortable with my positive status*. *I love myself*. *I understand that I can live peacefully whether I have a partner or not*. *I no longer stigmatise myself*. *I just feel I am as good as any other person*.*”* Post-course Interview Participant A10

## Discussion

This pilot study tested an intervention designed to help PLHIV to identify, control, and overcome the internal thoughts and beliefs that had previously created feelings of lowered self-worth, decreased agency, and negative mentality that contribute to self-stigma. Addressing our initial research question, *Does the intervention using IBSR*: *The Work of Byron Katie have potential to influence self-stigma*, the results clearly showed that after taking part in the intervention, the participants reported a significant reduction in levels of self-stigma. It is also noteworthy that many of the changes were maintained at 3-month follow-up, suggesting that the use of the intervention may facilitate longer-term control of self-stigma, with minimal refresher training. Several studies have indicated that reducing HIV self-stigma may improve access to care and wellbeing among PLHIV [70,71], and our current results suggest that after more complete development and testing, techniques from IBSR: The Work of Byron Katie might be employed in the future as an intervention to help achieve this.

Addressing our second research question, *Does the intervention have potential to influence stigma-associated states*, *such as fear around disclosure and depression*, we found that measures of both fears around disclosure, and depression, were significantly reduced following the IBSR: The Work of Byron Katie intervention. The reduction of stigma-associated states such as anxiety and depression is likely to improve wellbeing among PLHIV [45], and access to care is also likely to be improved if fears around disclosure can be overcome, particularly for those living in, or originating from, settings where HIV remains highly stigmatised [70,72]. In some cases, such as Life satisfaction, the reported scores were significantly improved at 1-month, but not 3-month follow-up. Conversely, some measures were not significantly improved at 1-month follow-up, but were significantly improved at 3 months, such as Feelings around being HIV-positive, or View of the future. This may have been due to certain aspects of the intervention taking longer to be internalised through repeated practice than others. If subsequent research shows these changes to be genuine effects of the intervention, supports such as refresher training could considered to help embed them more fully. It is not currently possible to judge the practical significance of the changes in scores for several reasons. First, the items are scored on arbitrary scales. It is not possible to assert that a specified change in, for example, life satisfaction score represents a 'real' change in life satisfaction. Furthermore, the score changes in this study are estimated from a small sample, and so are imprecise enough to caution against definitive claims. It is, however, encouraging that even in a small sample we were able to show statistically significant changes across a range of measures of wellbeing. Further well-considered research will elicit whether such changes can be replicated across a wider range of participants and contexts, and full attention should be given to understanding the ways in which those findings might translate into practical outcomes for the participants.

Beyond the patients’ self-reported reductions in measures such as self-stigma, fears around disclosure, and depression as empirical quantities, our qualitative observations contribute towards answering our third research question, *Does the intervention have potential to influence other areas of participants’ lives beyond HIV self-stigma*? The intervention’s participants reported a range of improvements across several aspects of their daily lives. Pre-intervention discussions illustrated the extent of participants’ negative outlook on life and lack of hope for the future, whereas post-intervention this had changed to a more positive dialogue, including making future plans, and improved self-image. Mannell *et al*., in their 2014 assessment of indicator frameworks for the HIV response, highlighted the need for greater provision for monitoring the social drivers and outcomes of the epidemic [47], and often the scales used to measure the effects of stigma in HIV define the *how much*, rather than the *why*. Our current results suggest transformative change simultaneously across multiple aspects of an individual’s life. This is thematically consistent with our previous research where multiple contributing factors and circumstances shaped core beliefs driving self-stigma among PLHIV, presenting a broad range of areas where targeted support could improve wellbeing and quality of life [12]. Using interventions incorporating IBSR: The Work of Byron Katie to address the underlying causes and outcomes in this holistic manner could therefore represent a useful means to reduce HIV self-stigma.

**Fig 3:**
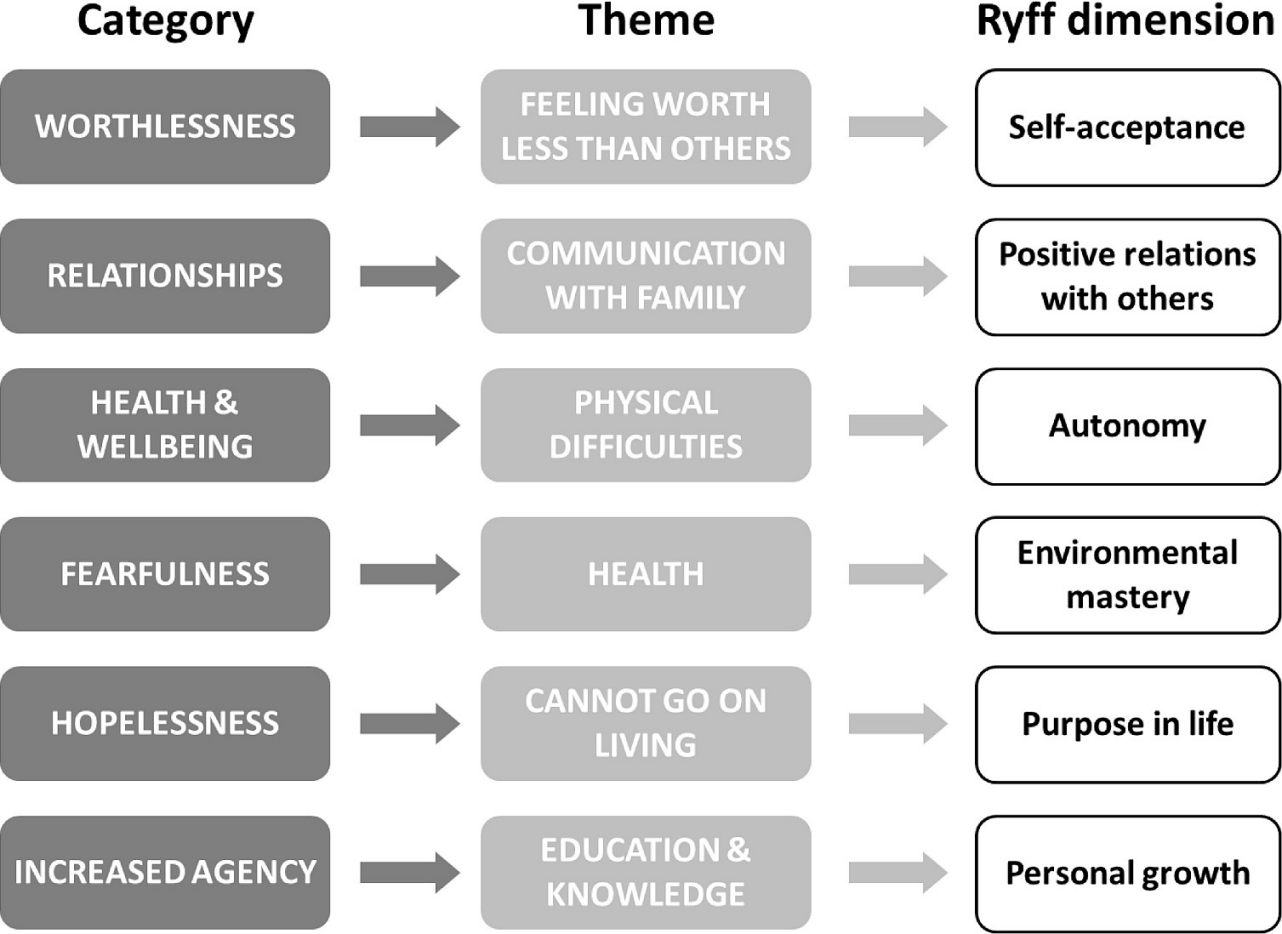
Alignment of themes from participant interviews with Ryff scales of wellbeing. Showing categories (left column, dark grey), and themes (middle column; light grey) which emerged during thematic analysis of participant interviews and focus group discussions. These broadly fell into the six dimensions of wellbeing (right column; unshaded), as proposed by Ryff and Keyes [73], suggesting that this scale may be of future utility in measuring outcomes following interventions that address HIV self-stigma.

It was most striking that the reported life changes reported after taking part in the intervention aligned in many cases with measures of psychological wellbeing, as proposed by Ryff and Keyes in 1995 [73] (Fig 3). The Ryff scales reflect dimensions such as self-acceptance, autonomy, life purpose, and personal growth and development–all of which were areas of improvement reported by this study’s participants during interviews. We would therefore argue that the incorporation of the Ryff scales into monitoring frameworks to capture greater information about changes to overall wellbeing, as opposed to simply measuring the negative outcomes such as the level of depression or the amount of stigma experienced, or quality of life at a single point in time, will hold significant utility in shaping responses and tracking intervention outcomes among PLHIV. This presents a timely opportunity to add a structured measurement of improvements in *positive attributes*, rather than focusing exclusively on removing negative attributes or problems, and expecting that the patient will be ‘well’ thereafter. In the current context of HIV prevention and care, this is especially relevant: good medication compliance and access to health services now enable the focus to shift away from merely controlling symptoms and remaining disease-free, towards promotion of wellbeing, maximizing productivity, and achieving life goals, within a normal 72–75 year life span [74,75]. However, this does not necessarily mean that PLHIV will be able to realise this potential; such a shift can only be fully achieved by providing access to health services in a manner that is free from external and internal stigma [76], and the ongoing persistence of self-stigma [18,33] indicates that further modes of support must now be sought and implemented.

Additionally, rather than simply managing the negative symptoms of self-stigma, our current results suggest that changes also occurred in overall wellbeing among participants, driving positive behavioural changes such as improved relationships with family, accessing self-improvement opportunities, and passing on knowledge to others. This intervention could therefore be a promising candidate for further investigation incorporating a more rigorous study design and expanded scope to test its potential effectiveness among larger and more diverse populations.

For the purposes of this pilot study we used internationally certified facilitators, although we recognised that this would be a challenge in terms of resources and sustainability. However, we believed that this was a necessary step to show that the IBSR methodology could be delivered and have the potential to influence self-stigma and other associated states within the urban Zimbabwean setting. Given that the intervention was relatively intensive, both in terms of duration, and its use of trained international facilitators, potential avenues for exploration could now include steps to reduce the length of the intervention, for example to distil it to the ‘minimum unit’ required. To this end, we are currently implementing and testing strategies to transition the intervention from its current ‘high-resource’ model towards one which can be suitably scaled up to reach the wider community, supported by locally-sourced and trained facilitators. Eleven local facilitators have now been trained in this method using a ‘train the trainers’ model, whereby the international facilitators adapted the training to be applicable in a low-resource setting. These steps are aiding in terms of retention of participants, as well as in building local expertise, moving away from reliance on bringing in international staff, and the effect of these changes is currently being investigated by CONNECT ZIST, Trócaire, The Work for Change, and RCSI.

Based on our current findings, we suggest that IBSR: The Work of Byron Katie should be investigated further as a novel, community-based intervention to help overcome the self-stigma that prevents PLHIV from pursuing life opportunities to the fullest extent.

### Study limitations

This was a small-scale pilot study which accessed participants from a single urban area in Zimbabwe, and did not contain a control group. As such, the results reported here cannot be generalized to the wider population. Future research in this area should incorporate larger pilot studies that feature a control group, before moving towards judicious use of best-practice methodologies including randomised controlled trials assigning participants either to the self-stigma intervention or an appropriately-considered comparator intervention.

Additionally, social stigma around minority sexual orientations in Zimbabwe precluded our asking participants to disclose their sexual orientations during recruitment, and we did not ask whether they had acquired HIV through heterosexual or same-gender sexual practices. Our findings, therefore, may not necessarily be generalizable across all sexual orientations. The characteristics and effects of stigma that affect different genders and sexualities are likely to vary, and future research should aim to investigate these differences in a sensitive manner, providing valuable insight into the complexities of stigma and self-stigma as they exert their effects on varied populations. This will be especially useful since it is likely that different groups will be responsive to different intervention modalities, therefore building the evidence-base in this area will yield important information for implementers.

We also acknowledge that IBSR is an intensive intervention which requires a significant time investment both from participants and skilled facilitators. In this first instance, we utilised facilitators sourced from outside Zimbabwe due to a lack of local facilitators, and to assure quality. Subsequent work is now focused on building a sustainable intervention that is delivered by a team of trained facilitators sourced from Zimbabwe, with the aim of it becoming fully Zimbabwean-led.

## Supporting information

S1 File Questionnaire administered to participants. (DOC)

S2 File Guides for pre- and post-programme Focus Group Discussions and post-programme interviews. (DOCX)

S3 File Questionnaire responses (Stata .dta file). (DTA)

S4 File Stata .do file of calculations performed. (DO)

S5 File List of categories, themes, and sub-themes. (XLSX)

S6 File Permission granted by EMH Swiss Medical Publishers to reproduce Fig 1. (PDF)

S7 File CONSORT flowchart. (DOC)

S8 File TREND checklist. (PDF)
