# Correction: 'We are the change'—An innovative community-based response to address self-stigma: A pilot study focusing on people living with HIV in Zimbabwe

**DOI:** 10.1371/journal.pone.0213465

**Published:** 2019-02-28

**Authors:** Nadine Ferris France, Stephen H.-F. Macdonald, Ronan M. Conroy, Patrick Chiroro, Deirdre Ni Cheallaigh, Masimba Nyamucheta, Bekezela Mapanda, Godsway Shumba, Dennis Mudede, Elaine Byrne

The third author's name is spelled incorrectly. The correct name is: Ronan M. Conroy. The correct citation is: Ferris France N, Macdonald SH-F, Conroy RM, Chiroro P, Ni Cheallaigh D, Nyamucheta M, et al. (2019) ‘We are the change’—An innovative community-based response to address self-stigma: A pilot study focusing on people living with HIV in Zimbabwe. PLoS ONE 14(2): e0210152. https://doi.org/10.1371/journal.pone.0210152

## References

[1] Ferris France N, Macdonald SH-F, Conroy RR, Chiroro P, Ni Cheallaigh D, Nyamucheta M, et al (2019) ‘We are the change’—An innovative community-based response to address self-stigma: A pilot study focusing on people living with HIV in Zimbabwe. PLoS ONE 14(2): e0210152 10.1371/journal.pone.0210152 30759114 PMC6373928

