# Supplementary material for: ‘We are the change’ - An innovative community-based response to address self-stigma: A pilot study focusing on people living with HIV in Zimbabwe
Source: PLoS One. 2019 Feb 13;14(2):e0210152. doi: 10.1371/journal.pone.0210152 (PMC6373928; doi:10.1371/journal.pone.0210152)
Supplement: S1 File — (DOC) [file pone.0210152.s001.doc]

**Self-stigma and HIV/AIDS**

**Baseline information**

To be used together with the Interviewer Guide that contains clear instructions for administering this questionnaire.

******************

**Participant ID:**

**Group:**

**Personal details**

1. What is your date of birth?: ________________________
2. How old are you? __________________________
3. Are you male or female? M____ F_____
4. Circle the highest grade or year of school that you completed.

Grade: 1 2 3 4 5 Form: 1 2 3 4 5 6 University:

1. Do you currently work for money?  ____________ Y/N
2. Are you married or in a committed relationship with a person you live with? ___________ Y?N
3. How many children, if any, do you have?

0 1 2 – 4 4+

1. How long have you been on treatment? __________ years
2. What ethnic group do you come from?________________
3. What is your religion

**Section A:**

|  | **Completely true** | **Mostly true** | **Mostly false** | **Completely false** |
| --- | --- | --- | --- | --- |
| 1. There are several people that you trust to help you solve problems |  |  |  |  |
| 1. There is no one that you feel comfortable talking to about intimate personal problems |  |  |  |  |
| 1. If you were sick and needed someone to take you to a doctor, I would have trouble finding someone |  |  |  |  |
| 1. If you needed a place to stay for a week because of an emergency, you could easily find someone who would put you up |  |  |  |  |
| 1. You fee that there is no one I can share your most private worries and fears with |  |  |  |  |
| 1. If you were sick, you could easily find someone to help you with your daily chores |  |  |  |  |
| 1. You feel a strong emotional bond with at least one other person |  |  |  |  |
| 1. When you need suggestions on how to deal with a personal problem, you know someone you can turn to |  |  |  |  |
| 1. If you needed an emergency loan there is someone you could get it from |  |  |  |  |
| 1. It would be difficult to find someone who would lend you their care for a few hours |  |  |  |  |
| 1. I have close relationships that provide me with a sense of emotional security and well-being |  |  |  |  |
| 1. You lack a feeling of intimacy with another person |  |  |  |  |

**Section B:**

|  | **0 days** | **1-2 days** | **3-4 days** | **5-7 days** |
| --- | --- | --- | --- | --- |
| 1. You were bothered by things that usually don’t bother you |  |  |  |  |
| 1. You did not feel like eating; your appetite was poor |  |  |  |  |
| 1. You felt that you could not shake off feeling down even with help from your family or friends |  |  |  |  |
| 1. You felt that you were just as good as other people |  |  |  |  |
| 1. You had trouble keeping your mind on what you were doing |  |  |  |  |
| 1. You felt depressed |  |  |  |  |
| 1. You felt that everything that you did was an effort |  |  |  |  |
| 1. You felt hopeful about the future |  |  |  |  |
| 1. You thought your life had been a failure |  |  |  |  |
| 1. You felt fearful |  |  |  |  |
| 1. Your sleep was restless |  |  |  |  |
| 1. You were happy |  |  |  |  |
| 1. You talked less than usual |  |  |  |  |
| 1. You felt lonely |  |  |  |  |
| 1. People were unfriendly |  |  |  |  |
| 1. You enjoyed life |  |  |  |  |
| 1. You had crying spells |  |  |  |  |
| 1. You felt sad |  |  |  |  |
| 1. You felt that people disliked you |  |  |  |  |
| 1. You could not get going |  |  |  |  |

**Section C:**

|  | **Strongly disagree** | **Slightly disagree** | **Slightly agree** | **Strongly agree** |
| --- | --- | --- | --- | --- |
| 1. I am actively involved in AIDS community issues |  |  |  |  |
| 1. It is difficult to tell other people about my HIV infection |  |  |  |  |
| 1. Being HIV positive makes me feel dirty |  |  |  |  |
| 1. There are many people in your life that you talk openly to about your HIV status |  |  |  |  |
| 1. You feel guilty that you are HIV positive |  |  |  |  |
| 1. You are ashamed that you are HIV positive |  |  |  |  |
| 1. In general, you feel proud of who you are |  |  |  |  |
| 1. You sometimes feel worthless because you are HIV positive |  |  |  |  |
| 1. It is your own fault that you are HIV positive |  |  |  |  |
| 1. Other than having HIV, you feel the same as everyone else |  |  |  |  |
| 1. You hide your HIV status from others |  |  |  |  |
| 1. More of your friends are HIV positive than HIV negative |  |  |  |  |

**Section D:**

**
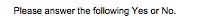
**


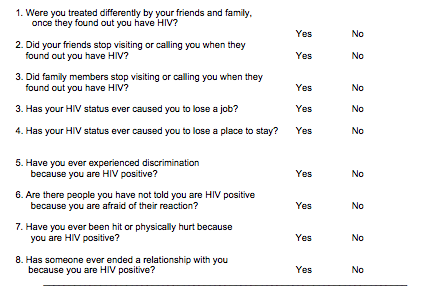


••••

**Section E:**

|  | **Strongly disagree** | **Slightly disagree** | **Slightly agree** | **Strongly agree** |
| --- | --- | --- | --- | --- |
| 1. You look forward to the future with hope and enthusiasm |  |  |  |  |
| 1. Sometimes you feel that everything is moving on ahead and leaving me behind |  |  |  |  |
| 1. You have great faith in future |  |  |  |  |
| 1. It is very hard for me to visualize what you will be like in five years |  |  |  |  |
| 1. The future seems vague and uncertain to me |  |  |  |  |

***Section F***

1. **The following questions ask about your overall function in the past 4 weeks:**

|  | **All**  **of the time** | **A lot**  **of the**  **time** | **Some**  **of the**  **time** | **A little**  **of the**  **time** | **None**  **of the**  **Time** |
| --- | --- | --- | --- | --- | --- |
| a. In the past 4 weeks, I've been satisfied  with my physical activity |  |  |  |  |  |
| b. In the past 4 weeks, I've been  physically limited in my ability to do  routine household chores |  |  |  |  |  |
| c. In the past 4 weeks, pain has limited  my ability to be physically active |  |  |  |  |  |
| d. In the past 4 weeks, I've been worried  about not being able to do my  job/routine daily activities as I have in  the past |  |  |  |  |  |
| e. In the past 4 weeks, I've felt that  having HIV has limited the amount of  work I can do at my job/routine daily  activities |  |  |  |  |  |
| f. In the past 4 weeks, I've been too tired  to be socially active |  |  |  |  |  |

1. **The following questions ask about your life satisfaction in the past 4 weeks:**

|  | **All**  **of the time** | **A lot**  **of the**  **time** | **Some**  **of the**  **time** | **A little**  **of the**  **time** | **None**  **Of the**  **Time** |
| --- | --- | --- | --- | --- | --- |
| 1. In the past 4 weeks, I've enjoyed living |  |  |  |  |  |
| 1. In the past 4 weeks, I've felt in control of my life |  |  |  |  |  |
| 1. In c. In the past 4 weeks, I've been satisfied with how socially active I am |  |  |  |  |  |
| 1. In the past 4 weeks, I've been   pleased with how healthy I've been |  |  |  |  |  |

1. **The following questions ask about your health worries in the past 4 weeks:**

|  | **All**  **of the time** | **A lot**  **of the**  **time** | **Some**  **of the**  **time** | **A little**  **of the**  **time** | **None**  **Of the**  **Time** |
| --- | --- | --- | --- | --- | --- |
| 1. In the past 4 weeks, I haven't been able to live the way I'd like to because I'm so worried about my health |  |  |  |  |  |
| 1. In the past 4 weeks, I've been worried about my CD4 count |  |  |  |  |  |
| 1. In the past 4 weeks, I've been worried about my viral load |  |  |  |  |  |
| 1. In the past 4 weeks, I've been worried about when I'm going to die |  |  |  |  |  |

**4. The following questions ask how you've felt about your HIV medications in the past 4 weeks:**

**Have you taken HIV medication in the past 4 weeks?**

**No -> -> -> -> -> -> Go to Section 5**

**Yes -> -> -> -> -> -> Continue with**

**Question 4a**

|  | **All**  **of the time** | **A lot**  **of the**  **time** | **Some**  **of the**  **time** | **A little**  **of the**  **time** | **None**  **Of the**  **Time** |
| --- | --- | --- | --- | --- | --- |
| 1. In the past 4 weeks, taking my   medicine has been a burden |  |  |  |  |  |
| 1. In the past 4 weeks, taking my   medicine has made it hard to live a  normal life |  |  |  |  |  |
| 1. In the past 4 weeks, taking my   medicine has caused unpleasant side  effects |  |  |  |  |  |
| d. In the past 4 weeks, I've been worried  about the effects my medicine may  have on my body |  |  |  |  |  |
| 1. In the past 4 weeks, I've been unsure   about why I'm taking medicine |  |  |  |  |  |

1. **The following questions ask how you've felt about being HIV positive in the past 4 weeks:**

|  | **All**  **of the time** | **A lot**  **of the**  **time** | **Some**  **of the**  **time** | **A little**  **of the**  **time** | **None**  **Of the**  **Time** |
| --- | --- | --- | --- | --- | --- |
| a. In the past 4 weeks, I've had regrets about the way I lived my life before knowing I had HIV |  |  |  |  |  |
| b. In the past 4 weeks, I've been angry about my past HIV risk behavior |  |  |  |  |  |

1. **The following questions ask about your disclosure worries in the past 4 weeks:**

|  | **All**  **of the time** | **A lot**  **of the**  **time** | **Some**  **of the**  **time** | **A little**  **of the**  **time** | **None**  **Of the**  **Time** |
| --- | --- | --- | --- | --- | --- |
| 1. In the past 4 weeks, I've limited what   I tell others about myself |  |  |  |  |  |
| 1. In the past 4 weeks, I've been afraid to tell other people that I have HIV |  |  |  |  |  |
| 1. In the past 4 weeks, I've been worried about my family members finding out that I have HIV |  |  |  |  |  |
| d. In the past 4 weeks, I've been worried  about people at my job/routine daily  activities finding out that I have HIV |  |  |  |  |  |
| e. In the past 4 weeks, I've been worried  that I'll lose my source of income if  other people find out that I have HIV |  |  |  |  |  |

1. **The following question ask about your sexual Experiences in the past 4 week:**

|  | **All**  **of the time** | **A lot**  **of the**  **time** | **Some**  **of the**  **time** | **A little**  **of the**  **time** | **None**  **Of the**  **Time** |
| --- | --- | --- | --- | --- | --- |
| 1. In the past 4 weeks, it has been   difficult to get sexually aroused |  |  |  |  |  |
| b. If you have had sexual experiences in the past 4 weeks, have you enjoyed it |  |  |  |  |  |
