# Supplementary material for: ‘We are the change’ - An innovative community-based response to address self-stigma: A pilot study focusing on people living with HIV in Zimbabwe
Source: PLoS One. 2019 Feb 13;14(2):e0210152. doi: 10.1371/journal.pone.0210152 (PMC6373928; doi:10.1371/journal.pone.0210152)
Supplement: S2 File — (DOCX) [file pone.0210152.s002.docx]

**We are the Change: IBSR programme to reduce self-stigma among people living with HIV and AIDS**

**Focus Group Discussion Guide (Pre-Programme)**

*Introductory notes:*

- Thank you for agreeing to take part in this programme on self-stigma called ‘We are the change’ inquiry-based stress reduction. We wanted to have a discussion with you today to understand some of your experiences about being HIV-positive and also ask you what you are hoping to get from the programme. If it’s okay with you, we will record the session so that we can type up the session later. All information will be confidential and anonymised and will not be shared with anyone outside of the research team.
- Introduce researchers
- Introduce participants

1. When you were first diagnosed with HIV, describe the feelings you had at the time?

Probe, shame, guilt, self-loathing, isolation, negative self-image

1. What do you think other people think about HIV-positive people?
2. What are your biggest fears related to your being HIV-positive?
3. What do you understand what we mean by self-stigma? What is your experience of self-stigma?
4. What do you hope to get from this course ‘We are the Change’ – inquiry-based stress reduction

**Focus Group Discussion Guide (Post-Programme)**

*Introductory notes: Thank you for agreeing to take part in this discussion to ask you to reflect on your experience of the programme. If it’s okay with you, we will record the session so that we can type up the session later. All information will be confidential and anonymised and will not be shared with anyone outside of the research team.*

1. If you had to describe what you got out of this course, what would you say?
2. What changes, if any did you experience in your daily life as a result of this programme?

Probe: among family, friends, work, social life

1. What changes did you experience, if any, about how you view and feel about yourself?
2. What did you learn about self-stigma?
3. What worked really well in this course?

Probe: length, topics covered, format, facilitators, homework, physical space, support, books/ipods

1. What did not work well in this course?

Probe: length, topics covered, format, facilitators, homework, physical space, support books/ipods

1. What other topics would you like to see covered in this course?
2. What would you do differently if we were doing the course again – what could be improved or changed?
3. How best could this course be delivered to others in Zimbabwe?

Probe: how can it be rolled out, local facilitators, within peer support groups etc.

1. What one word would you use to describe this course?

**One-on-one Interview Guide (post-programme)**

*Introductory notes: Thank you for agreeing to take part in this conversation to ask you to reflect on your experience of the programme. If It’s okay with you, we will record the session so that we can type up the session later. All information will be confidential and anonymised and will not be shared with anyone outside of the research team.*

1. If you had to describe what you got out of this course, what would you say?
2. What changes, if any did you experience in your daily life as a result of this programme? Probe: among family, friends, work, social life

*Please give specific examples*

1. What changes did you experience, if any, about how you view and feel about yourself?

After initial response, ask specifically about disclosure, about what it means to live with HIV, about sex and sexuality, about body, about illness and about death

1. What did you learn about self-stigma?
2. What worked really well in this course?

Probe: length, topics covered, format, facilitators, homework, physcial space, support, books/ipods

1. What did not work well in this course?

Probe: length, topics covered, format, facilitators, homework, physical space, support books/ipods

1. What would you do differently if we were doing the course again – what could be improved or changed?
2. What other topics would you like to see covered in the course?
3. How best could this course be delivered to others in Zimbabwe ?

Probe: how can it be rolled out, local facilitators, within peer support groups etc

10. What one word would you use to describe this course?
