# Supplementary material for: ‘We are the change’ - An innovative community-based response to address self-stigma: A pilot study focusing on people living with HIV in Zimbabwe
Source: PLoS One. 2019 Feb 13;14(2):e0210152. doi: 10.1371/journal.pone.0210152 (PMC6373928; doi:10.1371/journal.pone.0210152)
Supplement: S6 File — (PDF) [file pone.0210152.s006.pdf]

macdonaldez <macdonaldez@gmail.com>

Request to partially reproduce figure from our previous publication

office SMW <office@smw.ch> 26 October 2016 at 11:35
To: Steve H-F Macdonald <steve@globalhealth.ie>

Dear Steve

Thank you for your e-mail. We herewith grant you the licence to reprint the following items under CC-BY 4.0 license from our publication free of charge:

Figure 1
Author: Nadine Ferris France, Steve H. Macdonald, Ronan R. Conroy, Elaine Byrne, Chris Mallouris, Ian Hodgson, Fiona Larkan
Title: An unspoken world of unspoken things": a study identifying and exploring core beliefs underlying self-stigma among people living with HIV and AIDS in Ireland
Citation: Swiss Med Wkly. 2015;145:w14113. www.smw.ch

Acknowledgement to the publication with permission from EMH Swiss Medical Publishers Ltd. has to be made adjacent to the reproduced material with a credit line mentioning the complete citation.

Kind regards

Gisela

Gisela Wagner
Editorial office SMW

Swiss Medical Weekly
EMH Swiss Medical Publishers Ltd.
Farnsburgerstrasse 8
CH-4132 Muttenz
Switzerland
Phone +41 61 467 85 52
Fax +41 61 467 85 56
office@smw.ch
www.smw.ch
https://www.facebook.com/SwissMedicalWeekly
https://twitter.com/SwissMedWkly

Von: Steve H-F Macdonald [mailto:steve@globalhealth.ie]
Gesendet: Dienstag, 18. Oktober 2016 16:13
An: office SMW
Betreff: Re: Request to partially reproduce figure from our previous publication

Dear Gisela,
With apologies for yet another request, PLOS ONE have returned to me requesting that permission be granted with specific reference to the CC-BY 4.0 license, meaning that our new article will be available online to read, download, copy, distribute, and use in any way, with attribution, but without further permission. The summary of the license is here: https://creativecommons.org/licenses/by/4.0/
although I am sure you are already familiar with it.

Is it still permissible for us to reproduce the figure? If so, please would you provide an email with permission again, but specifically stating that the permission is granted to reproduce the partial figure under CC-BY 4.0 license? The figure would appear in our article as follows (the legend has been updated to include the specifications you requested in your previous email):

[Quoted text hidden]
