# Supplementary material for: ‘We are the change’ - An innovative community-based response to address self-stigma: A pilot study focusing on people living with HIV in Zimbabwe
Source: PLoS One. 2019 Feb 13;14(2):e0210152. doi: 10.1371/journal.pone.0210152 (PMC6373928; doi:10.1371/journal.pone.0210152)
Supplement: S7 File — (DOC) [file pone.0210152.s007.doc]

**
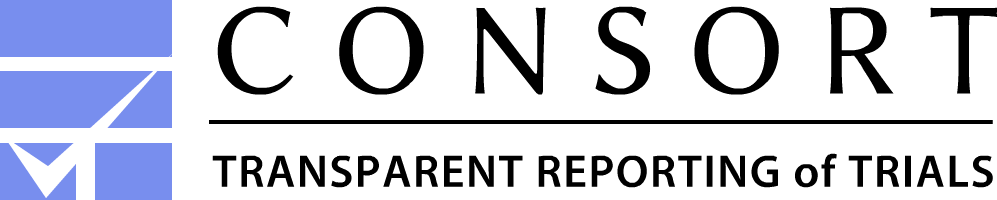
**

**CONSORT 2010 Flow Diagram**

**Allocation**

**Analysis**

**Follow-Up**

**Enrollment**

Assessed for eligibility (n=23)

Excluded (n=0)

♦  Not meeting inclusion criteria (n=0)

♦  Declined to participate (n=0)

♦  Other reasons (n=0)

Analysed (n=23)
♦ Excluded from analysis (give reasons) (n=0)

Lost to follow-up (give reasons) (n=0)

Discontinued intervention (give reasons) (n=0)

Allocated to intervention (n=23)

♦ Received allocated intervention (n=23)

♦ Did not receive allocated intervention (give reasons) (n=0)

Lost to follow-up (give reasons) (n=0)

Discontinued intervention (give reasons) (n=0)

Allocated to intervention (n=0)

♦ Received allocated intervention (n=0)

♦ Did not receive allocated intervention (give reasons) (n=0)

Analysed (n=0)
♦ Excluded from analysis (give reasons) (n=0)

Randomized (n=23)
